# Elucidating the effect of brewing temperature on the sensory quality of Longjing tea based on multi-scale molecular sensory science

**DOI:** 10.1016/j.fochx.2025.102635

**Published:** 2025-06-05

**Authors:** Sihan Deng, Qing-Qing Cao, Ying Gao, Weiwei Wu, Jian-Xin Chen, Fang Wang, Qian Zou, Fangxiang Xu, Xuefeng Cao, Weijiang Sun, Jun-Feng Yin, Yong-Quan Xu

**Affiliations:** a Tea Research Institute Chinese Academy of Agricultural Sciences, National Key Laboratory for Tea Plant Germplasm Innovation and Resource Utilization, 9 South Meiling Road, Hangzhou 310008, China; b College of Horticulture, Fujian Agriculture and Forestry University, Fuzhou 350002, China; c Hunan Chayue Culture Industry Development Group Co., LTD, 102-1, Building 8, Huayuan Hua Center, No. 36, Section 2, Xiangjiang Middle Road, Changsha 410118, China

**Keywords:** Brewing temperature, Metabolomics, Taste addition experiment, Molecular docking, Umami synergy

## Abstract

Longjing tea is popular due to its unique flavor characteristics, and the brewing process is of crucial importance in presenting its flavor. This study investigated how brewing temperature (BT) impacts Longjing tea's sensory and molecular profile. Sensory evaluations demonstrated that lower brewing temperatures (70–80 °C, with 70 °C optimal) enhanced umami taste while diminishing bitterness and astringency. LC-MS based metabolomics analysis revealed that the reduced BT enhanced umami taste by modulating free amino acid composition, while higher BT accelerated the leaching of flavonols and flavonol/flavone glycosides, exacerbating bitterness and astringency. Taste-addition experiments and molecular docking identified *L*-aspartic acid, *L*-theanine and *L*-arginine as key umami contributors, which synergistically enhanced umami intensity (1.77 to 6.83, *p* < 0.05) through stabilizing the Glu-T1R1/T1R3 complex and lowered binding free energy (−5.084 to −10.618 kcal/mol). These findings provided novel insights into the intricate relationship between BT and the flavor profile of Longjing tea.

## Highlights

• Brewing Longjing tea at 70–80 °C optimizes its desirable umami/umami-mellow tastes.
• Lower BT facilitates the extraction of low-MW compounds, enhancing the umami taste.
• 7-methylxanthine exhibits potential contribution to the umami taste of tea infusion.
• *L*-aspartic acid, *L*-theanine and *L*-arginine synergistically enhance umami taste.
• Sensory and molecular docking confirmed the synergistic effect of umami taste.

## 1 Introduction

Green tea is a traditional beverage in China that is highly precious by consumers for its unique flavor and a good deal health benefits, such as anti-cancer and antioxidant (Chen et al., 2018; Deng et al., 2023; Shan et al., 2023). As a representative variety of green tea, Longjing tea is famous for its fresh aroma and umami-mellow taste (Zhang et al., 2024). In recent years, with the enhancement of health awareness and the spread of tea culture, the economic value and market demand of Longjing tea have also increased (Hu et al., 2024).

The quality of Longjing tea is influenced by various factors, including the growing environment, harvesting season, processing methods and brewing conditions (Astill et al., 2001; Liu et al., 2024; Wang et al., 2022; Zhang et al., 2023). Among them, the brewing temperature (BT) plays a crucial role, significantly influencing the chemical composition and flavor of the tea infusion. During brewing process, BT influences the release of both volatile and non-volatile compounds in the tea leaves, finally impacting the color, aroma, taste, and other sensory characteristics of the tea infusion (Liu et al., 2024). Many studies have shown that an appropriate BT can not only improve the nutritional value of the tea but also boosts its antioxidant activity and the release of aromatic compounds (Pastoriza, Pérez-Burillo, & Rufián-Henares, 2016). When the BT increased from 60 °C to 90 °C, more catechins in the tea infusion underwent epimerization and the caffeine content increased significantly (Haihua et al., 2016). Cao et al. (2022) found that increasing the BT increased the extraction rate of amino acids, total polyphenols and caffeine. Zimmermann et al. (2011) also demonstrated that, in a short time, the efficiency of catechin extraction exceeded the degradation of catechins caused by high temperatures. In addition, the BT also significantly affected volatile compounds. Liu et al. (2021) reported that brewing black tea at 95 °C produced a distinct aromatic profile characterized by greener and sweeter notes, which could not be achieved at lower brewing temperature. The specific effects of different BTs on the flavor components of Longjing tea remain unclear, making it difficult to determine the optimal brewing conditions for the best flavor profile. In order to improve the brewing process and enhance the flavor quality of Longjing tea infusion, it is necessary to further elucidate the influence of BT on taste compounds. Additionally, exploring the relationship between these components and tea quality is critical to advancing our understanding of the key sensory attributes of Longjing tea.

Previous studies have indeed made significant progress in identifying principal umami-taste contributors in various teas. For instance, Kaneko et al. (2006) highlighted the crucial role of glutamic acid, gallic acid, theanine, theogallin and succinic acid as key contributors to the umami taste of Japanese matcha. Similarly, Yu et al. (2014) demonstrated that a deficiency in glutamic acid and aspartic acid led to a significant decrease in umami scores. More specifically for Longjing tea, Shan et al. (2024) comprehensively investigated its key umami contributors. Despite these important identifications of individual umami compounds, the synergistic mechanisms underlying overall umami perception in tea remain poorly understood. This is particularly true regarding the multi-ligand-receptor interaction patterns at the molecular level and the critical amino acid residues mediating these synergistic effects. This knowledge gap has hindered the development of comprehensive theoretical frameworks for elucidating umami perception mechanisms.

Therefore, this study aimed to investigate the impact of BTs on the sensory characteristics and key flavor compounds in Longjing tea. Different BTs were applied to Longjing tea, and the resulting infusions were comprehensively evaluated for their sensory attributes and key chemical profiles. The findings are intended to provide a deeper understanding of how BT influences Longjing tea quality and the underlying mechanisms of taste perception, ultimately aiding in the refinement of brewing recommendations.

## 2 Materials and methods

### 2.1 Materials

The premium Longjing (PL) and standard Longjing (SL) tea samples were supplied by the Tea Research Institute of the Chinese Academy of Agricultural Sciences. The main chemicals used in the study included pure water (Hang Zhou Wahaha Group Co., Ltd., Hangzhou, China); acetonitrile (HPLC, ≥99.9 %) and methanol (MeOH, HPLC grade, Merck, Darmstadt, Germany); gallic acid, formic acid, caffeine, (−)-epigallocatechin (EGC), (−)-epicatechin gallate (ECG), (−)-gallocatechin (GC), (−)-catechin gallate (CG), (−)-epigallocatechin gallate (EGCG), (−)-gallocatechin gallate (GCG), (−)-epicatechin (EC), (+)-catechin (C), glutamic acid, sodium hydroxide, monosodium glutamate (MSG), *L*-aspartic acid, *L*-arginine, as well as Folin-Ciocalteu's phenol from Shanghai Macklin Biochemical Co., Ltd. (Shanghai, China); *L*-theanine is provided by Shanghai Hope Industrial Co., Ltd. Additionally, decanoic acid ethyl ester (purity ≥99.5 %) was sourced from Shanghai Guo Yao Group Chemical Reagent Co., Ltd. (Shanghai, China).

### 2.2 Preparation of tea infusions

Tea samples were brewed with water at various temperatures (70, 80, 90, and 100 °C) and kept for 4 min. The dregs of tea were rapidly filtered out and the tea infusions were cooled down and incubated in a 45 °C water bath for subsequent sensory evaluation and experiments on non-volatile substances.

### 2.3 Analysis of chromatic parameters (color) of tea infusions

The color of tea infusions was measured using a Konica Minolta CM − 3500d spectrophotometer (Shanghai, China), by Xu et al. (2017) reported. The CIE *L*^⁎^*a*^⁎^*b*^⁎^ color space system was employed, where *L*^⁎^ denotes lightness on a scale from 0 (black) to 100 (white), *a*^⁎^ indicates the red (+)/green (−) chromaticity, and *b*^⁎^ represents the yellow (+)/blue (−) chromaticity. Measurements were taken under standardized D65 illuminant with a 10° standard observer configuration.

### 2.4 Sensory evaluation of tea infusions

#### 2.4.1 Quantitative descriptive analysis (QDA)

The tea infusions were maintained at 45 °C and assessed by an expert panel. A trained team of 9 panelists (4 men and 5 women) conducted the evaluation. Each panelist participated in a weekly flavor training program for a minimum 3 months, which included various reference standards and food items to improve their sensory abilities. The panelists evaluated the aroma and taste of each sample and scored it on a scale from 0 to 10 (0–2 means very weak; 2–4 means weak; 4–6 means moderate; 6–8 means strong; 8–10 means very strong) (Zeng et al., 2024).

#### 2.4.2 Taste addition experiments

The taste addition experiments were conducted following the methodology adapted from Shan et al. (2024). The three selected compounds (*L*-aspartic acid, *L*-theanine and *L*-arginine) were added to a 150 mg/L MSG (control group) based on their natural concentrations in tea infusions to evaluate their taste characteristics (Table S1). The experiment was divided into two parts. First, 20 trained panelists performed a paired comparison test to assess whether there were any umami taste differences between the control and experimental groups (mixed solutions with added compounds). Second, 5 professional assessors evaluated the experimental groups based on the umami rating criteria defined in the QDA. Each panelists scored independently. A 3-min interval was maintained between evaluations, during which panelists rinsed their mouths with purified water to prevent sensory fatigue and minimize carry-over effects.

No formal ethical committee or documentation is required for human sensory studies at the institute. However, appropriate measures were taken to protect participants' rights and privacy, including voluntary participation, full disclosure of study details and risks, written or verbal consent, non-disclosure of participant data, and the right to withdraw at any time. All samples used were food-grade to ensure safety. Informed consent was obtained from all participants prior to the sensory evaluation.

### 2.5 Analysis of the main non-volatile components

The total tea polyphenols were measured using the Folin-Ciocalteu reagent method. To 1 mL of the sample or gallic acid standard (0–100 mg/L) in a 10 mL volumetric flask, 5 mL of Folin-Ciocalteu reagent (10 %, *v/v*) was initially added. After a 5-min interval, 4 mL of Na_2_CO_3_ solution (7.5 %, *w/v*) was incorporated. The resulting mixture was then incubated at room temperature for 60 min before its absorbance was measured at 765 nm. The free amino acids were determined by the ninhydrin colorimetric method. 1 mL of sample or glutamic acid standard was reacted with phosphate buffer (pH = 8.0) and ninhydrin solution (2 %, *w*/*v*), shaken, and heated in a boiling water bath for 15 min. After cooling and dilution to 25 mL, solutions stood for 10 min before absorbance measurement at 570 nm against a distilled water blank (Cao et al., 2021; Deng et al., 2023).

The catechins, gallic acid and caffeine in infusions were analyzed using high-performance liquid chromatography (HPLC) with UV detector (Shimadzu, Tokyo, Japan), following Cao et al. (2021). The infusions were filtered using a 0.45 μm filter. The column used was a 4.6 mm × 250 mm, 5 μm Diamonsi™ C_18_ (Dikma Technologies Inc., Lake Forest, CA, USA), with the column temperature maintained at 35 °*C. mobile* phase A: 2 % acetic acid in water, mobile phase B: acetonitrile. The injection volume was 10 μL, the flow rate was at 1 mL/min, and the wavelength was 280 nm.

### 2.6 Compositional analysis by UHPLC-Q Exactive-mass spectroscopy

The untargeted metabolomic analysis was performed using ultra-high-performance liquid chromatography coupled with Quadrupole Exactive-Orbitrap mass spectrometry (Thermo Fisher Scientific, Rockford, IL, U.S.A.) following Wang et al. (2024) The study utilized a 1.8 μm ACQUITY UPLC HSS T3 column (2.1 mm × 100 mm, Waters, Milford, MA, U.S.A.) with column and autosampler temperatures of 40 °C and 10 °C. The gradient ranged from 5 % to 100 % ACN/0.1 % formic acid over 12 min. MS parameters included electrospray ionization in negative full scan mode, spray voltage of 3.1 kV and capillary temperature at 320 °C, scanning *m*/*z* 66.7 to 1000 Da.

Compound Discoverer™ 2.0 was used in conjunction with *mz*Cloud database (https://www.mzcloud.org) and ChemSpider database (https://www.chemspider.com/) to perform preliminary calibration (including deconvolution, peak extraction, peak alignment, etc.) and identification of detected peaks in different samples, and screening of key differential metabolites in combination with untargeted metabolomics analysis. The identification of these key differential metabolites was through Human Metabolome Database (https://www.hmdb.ca/). Metabolite abundances were relatively quantified by normalizing individual peak areas to the total integrated peak area across all detected metabolites in each sample.

### 2.7 Molecular docking

Given the unavailability of an experimental crystal structure for the human umami taste receptor (T1R1/T1R3 heterodimer), we predicted its three-dimensional architecture using AlphaFold 3 online platform (AF3, https://alphafoldserver.com/) based on the amino acid sequences of human T1R1 (UniProt: Q7RTX1) and T1R3 (UniProt: Q7RTX0) (Abramson et al., 2024; Cheng et al., 2023). The resulting model was subsequently validated using SAVESv6.0 (https://saves.mbi.ucla.edu/), including Ramachandran plot analysis.

Semi-flexible docking simulations were performed using AutoDock Vina 1.2.5 to study the interactions between single and multiple ligands, i.e., *L*-glutamate (Compound CID: 33032), *L*-aspartic acid (Compound CID: 5960), *L*-theanine (Compound CID: 439378), and *L*-arginine (Compound CID: 6322) with T1R1/T1R3-VFT (Eberhardt et al., 2021; Morris et al., 2009). The docking center coordinates were set as follows: center-x = −4, center-y = −5, center-z = 0, encompassed within a cubic grid box measuring 80 Å along each axis (x, y, z) with an exhaustiveness parameter set to 8. Binding affinity (kcal/mol) was used to evaluate docking results. The resulting docked conformations were ranked based on their binding affinity scores, and the pose with the lowest binding energy was selected for further analysis of interaction patterns (Yu et al., 2025).

### 2.8 Statistical analysis

The data are rendered as mean ± standard deviation (SD) from three independent replicates. The analysis was conducted using SPSS 23.0 (SPSS Inc., IBM Corporation, Armonk, NY, USA) with an analysis of variance (ANOVA). Additionally, a binomial test was used to compare the frequency differences. Partial least squares discriminant analysis (PLS-DA) was carried out in Simca-P (Version 13.0, MKS Umetrics AB, Umea, Sweden). The molecular docking results were visualized and analyzed using PyMOL (DeLano Scientific LLC, Palo Alto, USA).

## 3 Results and discussion

### 3.1 Effects of brewing temperature (BT) on the sensory quality of Longjing tea

**Fig. 1:**
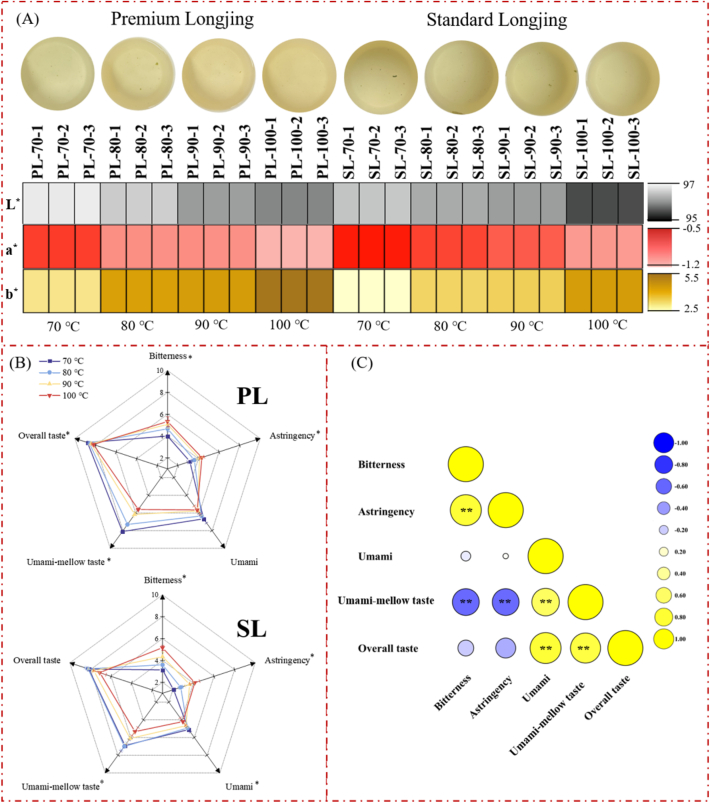
**Effects of brewing temperature on the sensory quality of Longjing tea.** The appearance color (A) and taste attributes (B) of Longjing tea infusion. (C) Correlation analysis between taste attributes. PL: Premium Longjing tea; SL: Standard Longjing tea. The symbols in the figure show the significance of differences between the means as tested by ANOVA followed by Duncan's test, ^⁎^ indicates *p* < 0.05. The heat map shows correlations between variables obtained by Pearson correlation analysis, ^⁎^ indicates *p* < 0.05, ^⁎⁎^ indicates *p* < 0.01.

As shown in Fig. 1A, the color of the infusions from different grades of Longjing tea with different BT were distinguishable. The color of the premium Longjing tea (PL) infusion was brighter than that of the standard Longjing tea (SL) infusion. The BT had a greater effect on PL than SL, especially on the lightness. This result was consistent with the color difference parameters. It was found that PL and SL brewed with 70 °C had the highest *L*^⁎^ and *a*^⁎^, lowest *b*^⁎^ values (*p* < 0.05), compared with the other temperature. The PL and SL investigated in this study represent distinct quality grades. This differentiation is primarily based on the tenderness of the fresh tea leaves utilized for their production, with PL typically being derived from younger and more tender leaves compared to SL.

The taste attributes of tea infusions were evaluated by an expert panel (Fig. 1B). With the increase of BT, the umami-mellow taste of PL significantly weakened, from 8.10 to 5.60 (*p* < 0.05), while bitterness (3.13 → 5.17, *p* < 0.05) and astringency (2.07 → 4.13, *p* < 0.05) of SL significantly enhanced. According to GB/T 14487–2017, umami-mellow is defined as “fresh and mellow”. Umami-mellow is recognized as an exceptional flavor for Longjing tea (Shan et al., 2025; Yan et al., 2025). The term refers to a rich, savory taste dominated by umami, balanced with a smooth and rounded depth. Thus, it's a multimodal oral sensation rather than the basic tastes (sour, sweet, bitter, salty, and umami), where canonical umami perception (mediated by T1R1/T1R3 receptors) serves as the foundational element. It was confirmed by the correlation analysis which revealed a highly significant positive correlation between umami and umami-mellow tastes (Fig. 1C; *r* = 0.62, *p* < 0.001). In addition, the overall taste of the tea infusion was closely related to both umami (*r* = 0.78, *p* < 0.001) and umami-mellow tastes (*r* = 0.74, *p* < 0.001).

In conclusion, tea infusions brewed at lower temperatures (70–80 °C) exhibited superior taste characteristics, but the underlying reasons need to be further explored. This phenomenon may be attributed to the differential release pattern of non-volatile compounds at different BT, which affects the overall flavor characteristics of tea infusion.

### 3.2 Non-volatile components in Longjing tea infusion under different BT

#### 3.2.1 Effect of BT on the major taste components in tea infusions

**Fig. 2:**
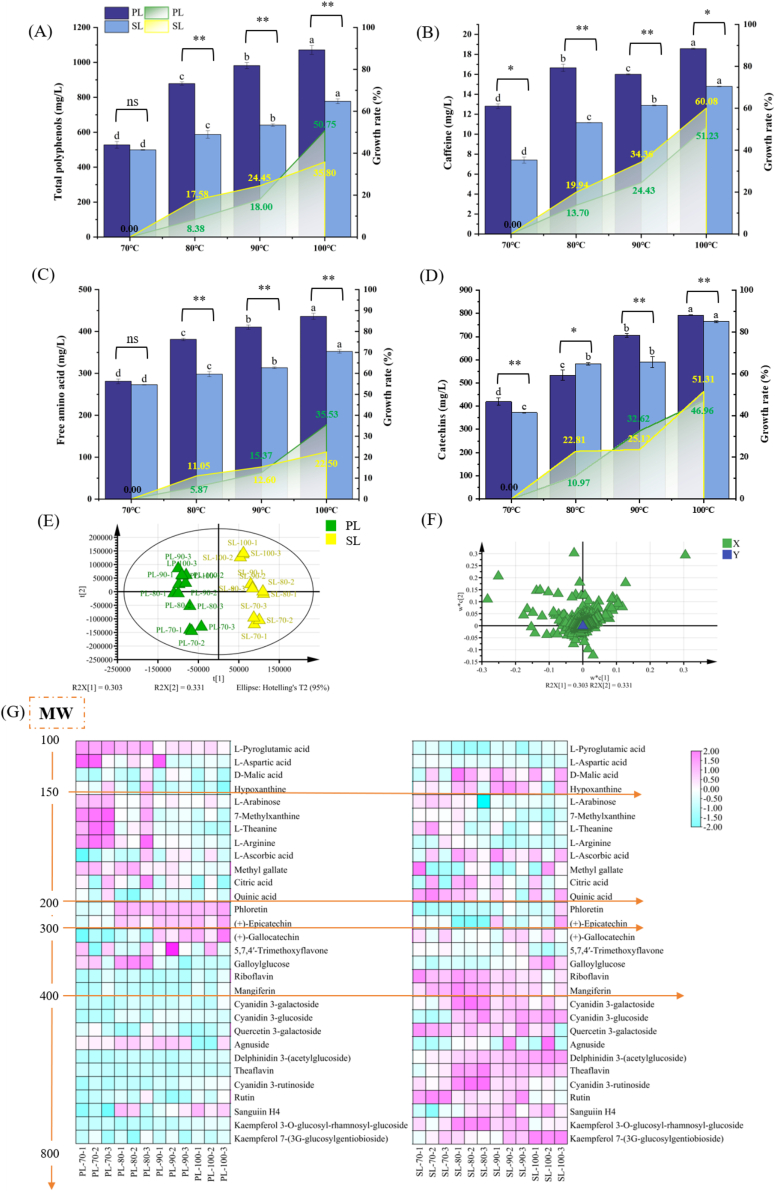
**The non-volatile components in Longjing tea infusion at different brewing temperatures.** The contents of total tea polyphenols (A), caffeine (B), free amino acid (C), and total catechins (D). A dual Y-axis is adopted to visualize their trends over brewing temperatures, the left axis represents the concentration (mg/L), while the right axis represents the growth rate (%). (E) ∼ (G) The PLS-DA analysis of the other non-volatile components. (E) The score scatter plots. (F) The loading scatter plots. (G) Heat map of the proportional contribution of the key differential component, and the components are ordered by ascending molecular weight (MW). PL: Premium Longjing tea; SL: Standard Longjing tea. The symbols in the figure show the significance of differences between PL and SL, ^⁎^ indicates *p* < 0.05, ^⁎⁎^ indicates *p* < 0.01. ^a, b, c, d^ Different letters above the same-color column indicate significant differences among different brewing temperatures (*p* < 0.05).

The contents and growth rates of the main taste components in Longjing tea infusions under different BT were shown in Fig. 2 & S1. The contents of total tea polyphenols, catechins, caffeine, and free amino acids were substantially higher in PL than SL. This finding was corroborated by our sensory evaluation, in which PL exhibited higher bitterness, astringency, umami and overall scores compared to SL. However, the rate of increase in bitterness and astringency was notably greater in SL compared to PL as BT rose. The bitterness and astringency score of PL increased by 33.25 % and 36.72 % from 70 °C to 100 °C, while the score in SL rose by 64.92 % and 99.98 %. Robichaud et al. (2010) believed that the intensities of bitterness and astringency increase linearly with the concentration of catechins, with bitterness increasing at a higher rate. In this study, the leaching rates of caffeine and catechins in SL were higher than in PL, with increases of 60.08 % and 51.31 %, respectively. Meanwhile, the flavor-contributing compounds in the tea infusion also interact with one another. Zhang et al. (2016) found that adding 2 mmol/L of caffeine significantly increased the bitterness of the infusion, an effect potentially attributed to EGC; this enhancement is likely due to an additive effect where caffeine's own intrinsic bitterness contributed to the overall perception alongside that of EGC, or possibly through synergistic interactions at the taste receptor level. Notably, when low concentrations of caffeine (0.5–1.0 mmol/L) were added, the astringency was reduced. Research indicates that when the BT increased (79.5 °C - 94.0 °C), the extraction rate constant and the extraction rate of caffeine both increased significantly (Spiro & Jago, 1982). Although catechins degrade at high temperature, their extraction rate is higher than the degradation rate under short-term high-temperature conditions (Zimmermann & Gleichenhagen, 2011).

In addition, it is commonly acknowledged that the concentration of free amino acids positively correlates with the umami/umami-mellow taste of tea (Wang et al., 2020). Intriguingly, our investigation revealed that for both PL and SL, the concentration of free amino acids increased significantly with BT, but the umami/umami-mellow taste actually weakened. This discrepancy could result from the variation in the extracted free amino acids composition, warranting further exploration into their roles in the taste.

#### 3.2.2 Effect of BT on the other non-volatile compounds

LC-MS analysis combined with multi-variate statistical analysis was employed to illustrate the effect on the other non-volatile compounds in tea infusions. Initially, 3539 metabolites were obtained, and 101 non-volatile metabolites were identified based on database matching (Table S2). The relative quantification results were utilized for PLS-DA analysis (Fig. 2E & F), which revealed clear separation between PL and SL groups (R^2^X(cum) =0.501, R^2^Y(cum) = 0.983, Q^2^(cum) = 0.962). The score plot showed that BT had a more pronounced effect on SL than on PL, particularly at BT exceeding 80 °C. In contrast, PL samples exhibited tighter spatial clustering, indicating greater compositional consistency. Using the PLS-DA model, 30 key differential compounds with a variable importance in the projection (VIP) > 1 were selected, and were arranged in heat maps based on their molar weight (MW) (Fig. 2G), including 8 organic acids, 14 flavonols and flavonol/flavone glycosides, 1 carbohydrate, 2 purines, 1 lipid, and 4 amino acids.

The compounds with MW of 100–200 g/mol, primarily free amino acids and organic acids, exhibited a higher relative content in PL (*p* < 0.05). Among which aspartic acid, glutamic acid, theanine and glutamine are well-established umami contributors in tea (Chen et al., 2020). Given that PL was processed from tender tea leaves, its elevated free amino acids contents likely explain its stronger umami/umami-mellow taste intensity compared to SL. It aligned with the findings mentioned above. Lower BT (70–80 °C) further enhanced the extraction efficiency of low-MW compounds in Longjing tea infusions. This phenomenon arises because smaller molecules diffuse more readily through the microporous structure of tea leaves, leading to faster leaching (Price & Spiro, 1985).

For the compounds with MW exceeded 400 g/mol, the majority were identified as flavonols and flavonol/flavone glycosides. Obviously, SL, made from more mature raw materials, contained much more these chemicals, except for Agnuside and Sanguiin H4 (*p* > 0.05), as reported previously (Tong, 2022). Delphinidin 3-(acetylglucoside) and theaflavin showed a substantial content differences (> 50 %) between PL and SL. Specifically, delphinidin 3-(acetylglucoside) decreased by 59.97 % in PL vs. SL (*p* < 0.0001), and theaflavin content was 53.55 % lower in PL (*p* < 0.0001). The former belongs to flavonol glycosides, a major subclass of flavonoids in tea leaves, contribute to the bitterness and astringency of tea infusion, along with caffeine and catechins (Ye et al., 2022). The astringency threshold of theaflavins (13–16 μmol/L) was much lower than that of catechins (190–930 μmol/L), and it was the key factors leading to more bitterness and astringency in SL (Scharbert & Hofmann, 2005).

**Fig. 3:**
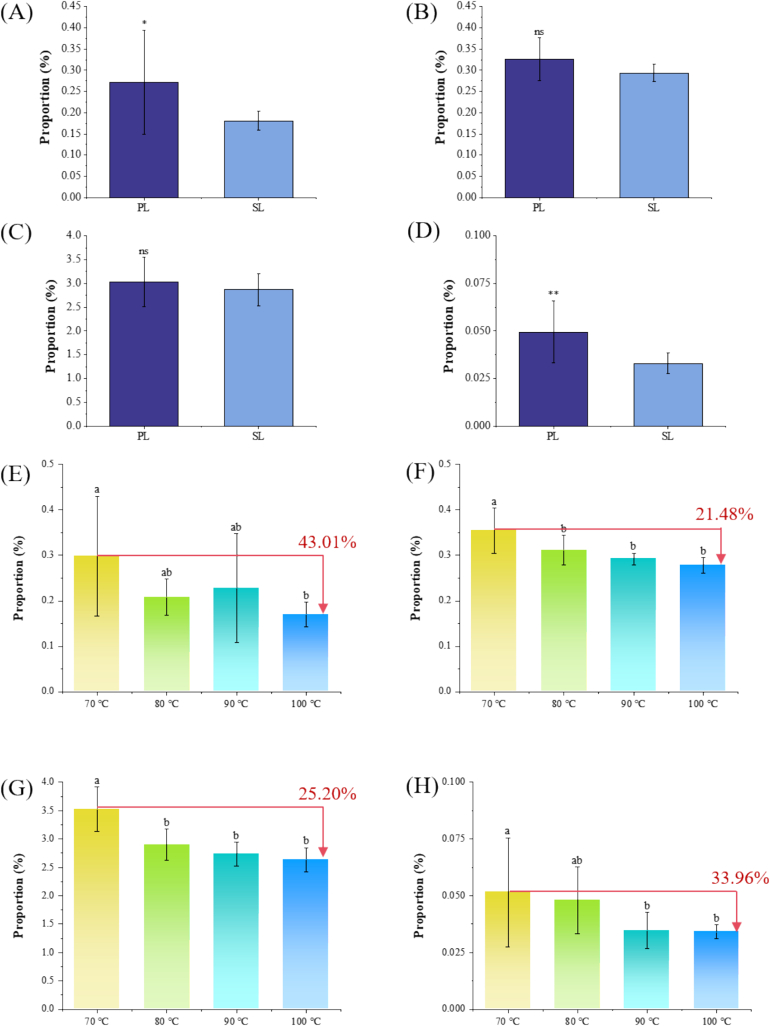
**The proportional contribution of the umami candidate compounds in the tea infusion of different tea samples**. (A) ∼ (D) and under different brewing temperatures (E) ∼ (H). (A) & (E): *L*-aspartic acid; (B) & (F): 7-methylxanthine; (C) & (G): *L*-theanine; (D) & (H): *L*-arginine. The numerical values in (E) ∼ (H) indicate the proportional reduction in the contribution of umami compounds. The symbols in the figure showed the significance of differences by a *t*-test, ^⁎^ indicates *p* < 0.05, ^⁎⁎^ indicates *p* < 0.01. Different letters on the columns indicated significant differences between the means as tested by ANOVA followed by Duncan's test (*p* < 0.05).

Besides, we conducted correlation analysis. *L*-aspartic acid (umami: *r* = 0.57, *p* < 0.01; umami-mellow taste: *r* = 0.53, *p* < 0.01), 7-methylxanthine (*r* = 0.61, *p* < 0.001; *r* = 0.76, *p* < 0.001), *L*-theanine (*r* = 0.47, *p* < 0.05; *r* = 0.79, *p* < 0.001), and *L*-arginine (*r* = 0.70, *p* < 0.001; *r* = 0.65, *p* < 0.001) were found to be correlated positively with both umami and umami-mellow taste. These compunds were further recognized as the potential key contributors for umami and umami-mellow taste of Longjing tea. Their relative contents reduced significantly (*p* < 0.05) with the elevated BT (Fig. 3E-H), among which *L*-aspartic acid decreased by 43.01 % from 70 °C to 100 °C, 7-methylxanthine 21.48 %, *L*-theanine 25.20 %, and *L*-arginine 33.96 %. Actually, *L*-aspartic acid, *L-*theanine, and *L*-arginine all play a certain role in umami taste of tea infusion (Hiranpradith et al., 2023; Kaneko et al., 2006; Liu et al., 2024). *L-*theanine accounts for more than 50 % of the total amino acids in green tea leaves, and is believed to be a key contributor to the umami taste (Kaneko et al., 2006). *L*-arginine was reported to enhance the perception of umami (Chaudhari & Roper, 2010). However, there have been no reports on the contribution of 7-methylxanthine to the umami taste of tea infusion so far.

### 3.3 Taste addition experiment of umami-contributors

The taste contribution of the four selected potential key contributors were assessed using a taste addition experiment. Unfortunately, due to the water insolubility of 7-methylxanthine, only *L*-aspartic acid, *L*-theanine and *L*-arginine were included in the subsequent analysis, while the umami contribution of 7-methylxanthine could not be confirmed (Table S1). The sensory analysis results showed that although these compounds exhibited umami, the perceived intensities were relatively low, ranging from 1.43 to 2.13 (Table S3).

**Fig. 4:**
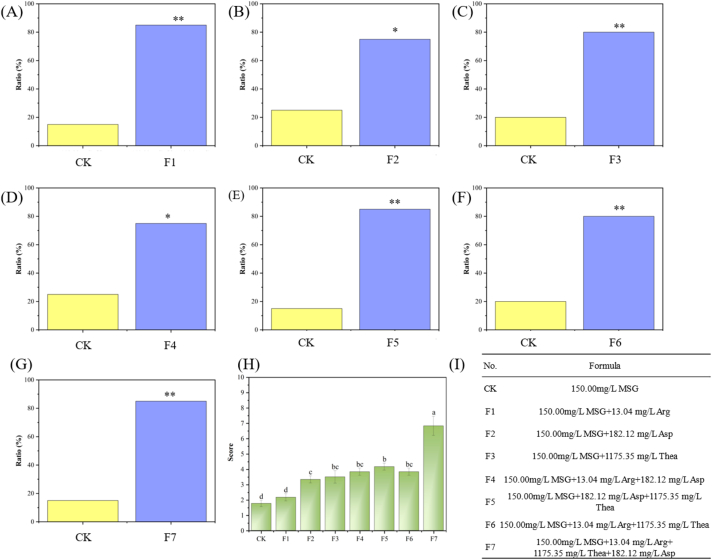
**Validation of umami synergistic effects through taste addition experiments.** (A-G) Analysis of paired comparison tests (*n* = 20); (H) Analysis of QDA (*n* = 5); (I) Formula of taste addition experiments. Arg: *L*-aspartic acid; Asp: *L*-aspartic acid; Thea: *L*-theanine. The marks in the (A) ∼ (G) showed the significance of differences by a binomial test, ^⁎^ indicates *p* < 0.05, ^⁎⁎^ indicates *p* < 0.01. Different letters on the columns indicated significant differences between the means as tested by ANOVA followed by Duncan's test (*p* < 0.05).

Using a 150 mg/L MSG solution as the control (CK), the potential contributors were added to further investigate their effects on umami taste. The paired comparison tests revealed significant sensory differences between the mixed solutions at varying formulas (F1-F7) and CK (Fig. 4A-G). This is consistent with the results of Shan et al. (2024). QDA results (Fig. 4H) further demonstrated the addition of the three candidate compounds to the CK resulted in a noticeable enhancement of umami, with *L*-theanine showing the most significant effect, achieving 3.50 ± 0.41. The addition of their ternary mixtures resulted in a continuous increase in the umami score, and when the quaternary mixture was added, the score reached 6.83 ± 0.62, showing an increase of 5.06 points compared to CK. This suggests that these three candidate compounds are not only umami-taste contributors, but also exhibit a synergistic effect in enhancing umami taste.

### 3.4 Molecular docking analysis of the binding interactions between key umami-taste contributors and taste receptors

**Fig. 5:**
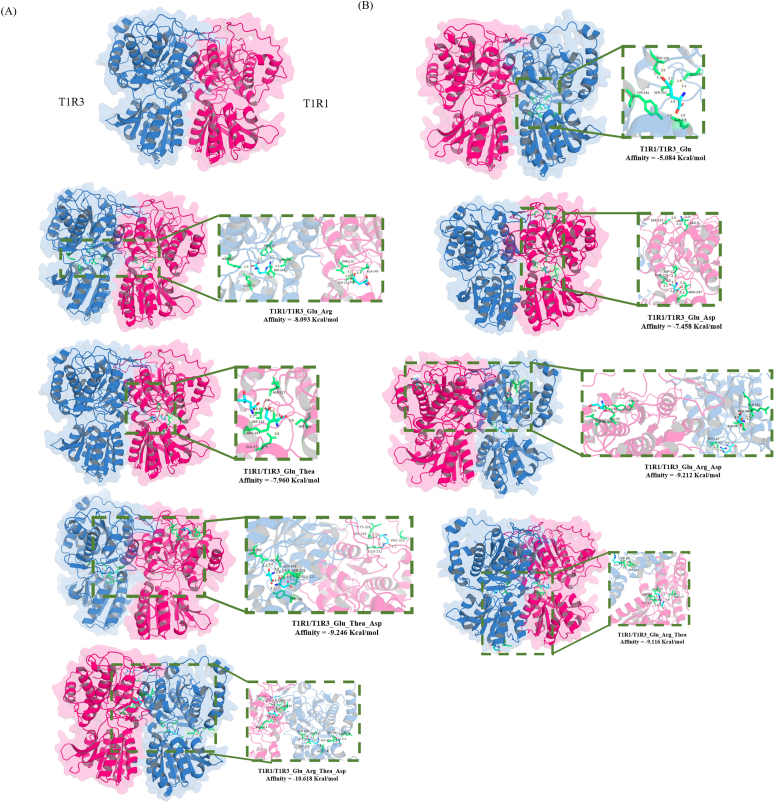
**The molecular docking between the key umami contributors with umami receptor T1R1/T1R3.** (A) Prediction structure of umami receptor T1R1/T1R3. (B) Molecular docking results of different formula. The red conformation represented T1R1, while the blue conformation represents T1R3. (For interpretation of the references to color in this figure legend, the reader is referred to the web version of this article.)

To elucidate the molecular mechanism underlying the synergistic umami enhancement of the three umami-taste contributors, we conducted molecular docking using Autodock Vina. Human umami taste receptor (i.e., T1R1/T1R3 heterodimer) were modeled on AlphaFold 3 (Fig. 5A) and its structural validity was confirmed via the Ramachandran plot analysis. It showed that 95.39 % of the amino acid residues fell within the reasonable zone, with 93.30 % were in the “most favoured” regions and 6.70 % in the “additional allowed” regions (Fig. S4). shown as Fig. 5B (Ruan et al., 2021).

While MSG served as the control in sensory experiments, *L*-glutamic acid was selected for molecular docking studies to precisely validate its interaction with T1R1/T1R3 (Yasumatsu et al., 2015). Binding affinity serves as a key indicator of docking efficiency and complex stability, with lower values indicating higher affinity and a more stable complex conformation (Yu et al., 2025). Compared to Glu with a binding energy of only −5.084 Kcal/mol, the binary ligand complex exhibited decreased binding energy ranging from −7.458 Kcal/mol to −8.093 Kcal/mol after adding *L*-arginine. Despite low umami intensity (2.17 ± 0.24) for *L*-arginine alone, 85 % of participants detected significant its umami enhancement in paired testing. Among the ternary ligands, F5 (150.00 mg/L MSG + 182.12 mg/L Asp +1175.35 mg/L Thea) exhibited the lowest affinity at −9.246 Kcal/mol, while the quaternary ligand showed the lowest affinity at −10.618 Kcal/mol above all formulas, consistent with our previous sensory evaluation results.

In the single-ligand docking simulation, Glu interacted with T1R1/T1R3 involving only four amino acid residues, forming a total of four hydrogen bonds. Multi-ligand docking presented significant increases in both the number of interacting amino acid residues (54 vs. 4 in single-ligand docking) and hydrogen bonds (79 vs. 4), indicating tighter intermolecular interactions. This suggests that multiple ligands (*L*-aspartic acid, *L*-theanine and *L*-arginine) can simultaneously occupy the receptor's binding pocket, where they may generate synergistic interactions that expand the binding region. This phenomenon shows similarity to the established mechanism of small-molecule synergy, where multiple ligands binding to the same target may integrate interactions to form complexes with enhanced affinity (Yu et al., 2025). This provides a potential molecular basis for the umami synergy observed in our sensory validation. Among these, residues Asp-117, Thr-119, Ser-142, Ser-143 and Ser-120 were identified as high-frequency binding sites, appearing 3, 3, 3, 5 and 5 times, respectively, playing a crucial role in the interactions. Liang et al. (2022) have reported that the Glu, Ser, Arg, Asp and His residues paved the way for molecular docking and were the key residues for the binding of umami peptides to T1R1/T1R3. And all hydrogen bond distances were notably short, ranging from 2.1 to 3.3 Å, indicating a strong binding affinity of the four compounds to the taste receptor pocket, which led to a stable complex conformation (Shiyan et al., 2021). Dang et al. (2019) proposed a mechanism for synergistic umami perception, suggesting that MSG initially binds to the T1R1 pocket. Upon MSG binding, ligand-induced dimerization leads to a pronounced expansion of the orthosteric pocket in the T1R1/T1R3 receptor, reportedly more than doubling its volume from approximately 534 Å^3^ to 1136 Å^3^. This expanded cavity enables the receptor to accommodate umami-enhancing peptides more readily. When multiple ligands bind the receptor concurrently, shared interaction sites play a core role in stabilizing the multicomponent complex (Zhang et al., 2008). These interactions stabilize activating conformational changes in the receptor, thereby enhancing umami perception through a synergistic effect. These findings illustrated that *L*-aspartic acid, *L*-theanine, and *L*-arginine synergistically enhanced umami perception by stabilizing the Glu-T1R1/T1R3 complex.

## 4 Conclusions

In general, this study explored the impact of different BT on the multi-scale molecular sensory profile of various grades of Longjing tea. The sensory evaluation results indicated that SL was more prone to BT, and lower BT enhanced the umami/umami-mellow taste, while reduced the bitterness and astringency of tea infusion. Furthermore, we found an interesting phenomenon between the BT and the leaching of free amino acids: when BT was 70 °C, the umami/ umami-mellow taste score was the highest, but the concentration of free amino acids was significantly lower than the sample brewed at 100 °C. LC-MS analysis indicated that the proportion of selected differential amino acids decreased with increasing temperature, which helped explain the changes observed in sensory evaluation. Additionally, the higher and faster leaching ratio of flavonols and flavonol/flavone glycosides of SL caused by the lower tenderness of its raw materials, contributed to the quicker increase in bitterness and astringency as BT rose compared to PL. Taste addition experiment and molecular docking further confirmed that three key umami-taste contributors can spontaneously bind to the umami receptor. Compared to single ligands, multiple ligands exhibit more stable conformations upon binding, indicating synergistic effects between them. These findings offer new insights into the complex relationship between the BT, sensory characteristics, and molecular chemistry of Longjing tea, providing practical significance for optimizing brewing techniques to enhance flavor quality across different tea grades. Additionally, they offer theoretical support for understanding the mechanism of umami synergism.

## CRediT authorship contribution statement

**Sihan Deng:** Writing – original draft, Visualization, Methodology, Investigation, Formal analysis, Data curation. **Qing-Qing Cao:** Writing – review & editing, Supervision, Resources, Methodology, Data curation. **Ying Gao:** Writing – review & editing, Visualization, Software, Resources. **Weiwei Wu:** Software. **Jian-Xin Chen:** Methodology. **Fang Wang:** Writing – review & editing. **Qian Zou:** Writing – review & editing. **Fangxiang Xu:** Data curation. **Xuefeng Cao:** Software. **Weijiang Sun:** Supervision. **Jun-Feng Yin:** Investigation, Data curation. **Yong-Quan Xu:** Writing – review & editing, Project administration, Investigation, Formal analysis, Data curation, Conceptualization.

## Declaration of competing interest

The authors declare that they have no known competing financial interests or personal relationships that could have appeared to influence the work reported in this paper.

## Data Availability

Data will be made available on request.

## Appendix A Supplementary data

Supplementary material. Image 1
