# Supplementary material for: Elucidating the effect of brewing temperature on the sensory quality of Longjing tea based on multi-scale molecular sensory science
Source: Food Chem X. 2025 Jun 5;28:102635. doi: 10.1016/j.fochx.2025.102635 (PMC12181020; doi:10.1016/j.fochx.2025.102635)
Supplement: Supplementary file 1 — Supplementary material. [file mmc1.docx]

**Elucidating the effect of brewing temperature on the sensory quality of Longjing tea based on multi-scale molecular sensory science**

Sihan Deng ^1, 2^, Qing-Qing Cao ^1*^, Ying Gao ^1^, Weiwei Wu ^2^, Jian-Xin Chen ^1^, Fang Wang ^1^, Qian Zou ^3^, Fangxiang Xu ^3^, Xuefeng Cao ^3^, Weijiang Sun ^2^, Jun-Feng Yin ^1^, Yong-Quan Xu ^1*^

^1^ *Tea Research Institute Chinese Academy of Agricultural Sciences, National Key Laboratory for Tea Plant Germplasm Innovation and Resource Utilization, 9 South Meiling Road, Hangzhou 310008, China*

^2^ *College of Horticulture, Fujian Agriculture and Forestry University, Fuzhou, 350002, China*

^3^ *Hunan Chayue Culture Industry Development Group Co., LTD, 102-1, Building 8, Huayuan Hua Center, No. 36, Section 2, Xiangjiang Middle Road, Changsha, 410118, China*

**Corresponding Authors**

* Yong-Quan Xu, Tel: +86-571-86017633. Fax: +86 571 86650056. Email: yqx33@126.com.

* Qing-Qing Cao, Tel: +86-571-86650594. Fax: +86 571 86650056. Email: caoqingqing@tricaas.com.

Table S1 Formula of taste addition experiments.

| No. | Formula |
| --- | --- |
| CK | 150.00mg/L MSG |
| F1 | 150.00mg/L MSG + 13.04 mg/L Arg |
| F2 | 150.00mg/L MSG + 182.12 mg/L Asp |
| F3 | 150.00mg/L MSG + 1175.35 mg/L Thea |
| F4 | 150.00mg/L MSG + 13.04 mg/L Arg + 182.12 mg/L Asp |
| F5 | 150.00mg/L MSG + 182.12 mg/L Asp + 1175.35 mg/L Thea |
| F6 | 150.00mg/L MSG + 13.04 mg/L Arg + 1175.35 mg/L Thea |
| F7 | 150.00mg/L MSG + 13.04 mg/L Arg + 1175.35 mg/L Thea + 182.12 mg/L Asp |

Notes: MSG: Monosodium Glutamate; Arg: *L*-aspartic acid; Asp: *L*-aspartic acid; Thea: *L*-theanine.

Table S2 The proportion of the relative content of non-volatile compounds to the total relative content in Longjing tea with different BT.

| No. | Retention time  (min) | | Accurate mass | Metabolomics identification | Molecular formula | MS^2^ fragments | Categories | PL-70℃ | PL-80℃ | PL-90℃ | PL-100℃ | SL-70℃ | SL-80℃ | SL-90℃ | SL-100℃ |
| --- | --- | --- | --- | --- | --- | --- | --- | --- | --- | --- | --- | --- | --- | --- | --- |
| 1 | 0.87 | 174.20 | | L-Arginine | C6H14N4O2 | 85, 156, 173 | Amino acids | 0.070 ± 0.004 | 0.056 ± 0.015 | 0.042 ± 0.001 | 0.036 ± 0.003 | 0.030 ± 0.001 | 0.040 ± 0.003 | 0.028 ± 0.002 | 0.032 ± 0.002 |
| 2 | 0.92 | 334.07 | | Glycerophosphoinositol | C9H19O11P | 78, 241, 245 | Lipids | 0.021 ± 0.001 | 0.026 ± 0.004 | 0.025 ± 0.002 | 0.022 ± 0.002 | 0.033 ± 0.002 | 0.022 ± 0.001 | 0.043 ± 0.002 | 0.028 ± 0.002 |
| 3 | 0.93 | 97.98 | | Phosphate | H3O4P | 78, 96 | Organic acid | 0.125 ± 0.005 | 0.110 ± 0.002 | 0.102 ± 0.004 | 0.112 ± 0.007 | 0.135 ± 0.004 | 0.099 ± 0.005 | 0.113 ± 0.001 | 0.099 ± 0.005 |
| 4 | 0.94 | 166.14 | | 7-Methylxanthine | C6H6N4O2 | 147, 165 | Purines | 0.399 ± 0.010 | 0.326 ± 0.030 | 0.292 ± 0.007 | 0.284 ± 0.017 | 0.310 ± 0.002 | 0.298 ± 0.022 | 0.293 ± 0.015 | 0.272 ± 0.011 |
| 5 | 0.96 | 89.99 | | Oxalic acid | C2H2O4 | 88 | Organic acids | 0.330 ± 0.020 | 0.347 ± 0.012 | 0.321 ± 0.008 | 0.349 ± 0.026 | 0.336 ± 0.013 | 0.345 ± 0.016 | 0.326 ± 0.014 | 0.333 ± 0.024 |
| 6 | 0.96 | 136.11 | | Hypoxanthine | C5H4N4O | 99, 135 | Purines | 0.369 ± 0.016 | 0.362 ± 0.032 | 0.339 ± 0.009 | 0.323 ± 0.015 | 0.369 ± 0.008 | 0.382 ± 0.025 | 0.414 ± 0.008 | 0.368 ± 0.033 |
| 7 | 0.96 | 133.10 | | L-Aspartic acid | C4H7NO4 | 71, 88, 115 | Amino acids | 0.391 ± 0.110 | 0.237 ± 0.031 | 0.270 ± 0.143 | 0.189 ± 0.012 | 0.206 ± 0.005 | 0.180 ± 0.008 | 0.187 ± 0.005 | 0.151 ± 0.017 |
| 8 | 0.97 | 230.02 | | D-Xylulose 1-phosphate | C8H2N6O3 | 78, 96 | Carbohydrates | 0.093 ± 0.004 | 0.086 ± 0.003 | 0.086 ± 0.004 | 0.095 ± 0.005 | 0.111 ± 0.010 | 0.087 ± 0.007 | 0.089 ± 0.010 | 0.095 ± 0.006 |
| 9 | 0.98 | 118.03 | | Succinic acid | C4H6O4 | 73, 99, 116 | Organic acids | 0.077 ± 0.007 | 0.078 ± 0.003 | 0.074 ± 0.001 | 0.076 ± 0.006 | 0.079 ± 0.006 | 0.067 ± 0.006 | 0.064 ± 0.005 | 0.071 ± 0.005 |
| 10 | 0.98 | 290.04 | | D-Sedoheptulose 7-phosphate | C7H15O10P | 78, 96, 271, 289 | Carbohydrates | 0.082 ± 0.004 | 0.074 ± 0.003 | 0.076 ± 0.005 | 0.087 ± 0.005 | 0.099 ± 0.009 | 0.075 ± 0.007 | 0.077 ± 0.009 | 0.085 ± 0.006 |
| 11 | 0.99 | 263.06 | | Ascorbalamic acid | C10H9N5O4 | 59, 71, 96, 101 | Amino acids | 0.088 ± 0.004 | 0.080 ± 0.009 | 0.080 ± 0.005 | 0.081 ± 0.006 | 0.097 ± 0.003 | 0.086 ± 0.005 | 0.077 ± 0.005 | 0.086 ± 0.005 |
| 12 | 1.00 | 143.02 | | 6-Methylquinoline | C10H9N | 91，142 | Alkaloids | 0.006 ± 0.000 | 0.005 ± 0.000 | 0.006 ± 0.001 | 0.005 ± 0.000 | 0.008 ± 0.001 | 0.007 ± 0.001 | 0.007 ± 0.001 | 0.008 ± 0.001 |
| 13 | 1.00 | 192.17 | | Quinic acid | C7H12O6 | 85, 93, 191 | Organic compounds | 0.694 ± 0.038 | 0.615 ± 0.034 | 0.667 ± 0.009 | 0.664 ± 0.026 | 0.803 ± 0.018 | 0.736 ± 0.036 | 0.695 ± 0.049 | 0.743 ± 0.053 |
| 14 | 1.01 | 304.04 | | Taxifolin | C15H12O7 | 177, 191, 231 | Flavanonols | 0.077 ± 0.005 | 0.064 ± 0.006 | 0.060 ± 0.001 | 0.064 ± 0.001 | 0.084 ± 0.003 | 0.068 ± 0.002 | 0.062 ± 0.005 | 0.059 ± 0.002 |
| 15 | 1.01 | 312.32 | | 5, 7, 4′-Trimethoxyflavone | C18H16O5 | 179, 311 | Flavonoids | 0.098 ± 0.033 | 0.097 ± 0.021 | 0.129 ± 0.041 | 0.101 ± 0.021 | 0.102 ± 0.003 | 0.093 ± 0.002 | 0.097 ± 0.009 | 0.075 ± 0.006 |
| 16 | 1.02 | 324.04 | | 5′-UMP | C9H13N2O9P | 78, 96, 111 | Nucleosides | 0.067 ± 0.001 | 0.064 ± 0.003 | 0.063 ± 0.003 | 0.063 ± 0.006 | 0.064 ± 0.003 | 0.067 ± 0.005 | 0.065 ± 0.003 | 0.067 ± 0.004 |
| 17 | 1.03 | 180.06 | | D-Glucose | C6H12O6 | 59, 179 | Carbohydrates | 0.177 ± 0.007 | 0.154 ± 0.026 | 0.151 ± 0.009 | 0.151 ± 0.013 | 0.183 ± 0.008 | 0.161 ± 0.007 | 0.152 ± 0.003 | 0.147 ± 0.006 |
| 18 | 1.03 | 133.04 | | Iminodiacetic acid | C4H7NO4 | 88, 114, 132 | Amino acids | 0.066 ± 0.039 | 0.064 ± 0.025 | 0.047 ± 0.020 | 0.039 ± 0.035 | 0.020 ± 0.004 | 0.062 ± 0.037 | 0.052 ± 0.044 | 0.025 ± 0.008 |
| 19 | 1.04 | 150.13 | | L-Arabinose | C5H10O5 | 75, 131, 149 | Carbohydrates | 0.224 ± 0.004 | 0.195 ± 0.017 | 0.188 ± 0.005 | 0.187 ± 0.009 | 0.207 ± 0.007 | 0.136 ± 0.079 | 0.174 ± 0.004 | 0.169 ± 0.007 |
| 20 | 1.06 | 347.06 | | Adenosine 5'-monophosphate | C10H14N5O7P | 78, 96 | Nucleosides | 0.053 ± 0.007 | 0.048 ± 0.002 | 0.045 ± 0.004 | 0.047 ± 0.004 | 0.055 ± 0.007 | 0.052 ± 0.002 | 0.051 ± 0.004 | 0.048 ± 0.003 |
| 21 | 1.10 | 134.09 | | D-Malic acid | C4H6O5 | 71, 115 | Organic compounds | 0.655 ± 0.065 | 0.692 ± 0.100 | 0.723 ± 0.063 | 0.643 ± 0.065 | 0.733 ± 0.102 | 0.944 ± 0.108 | 0.872 ± 0.086 | 0.888 ± 0.097 |
| 22 | 1.12 | | 176.12 | L-Ascorbic acid | C6H8O6 | 71, 115, 175 | Organic compounds | 1.086 ± 0.130 | 1.253 ± 0.142 | 1.286 ± 0.085 | 1.258 ± 0.081 | 1.286 ± 0.106 | 1.436 ± 0.088 | 1.440 ± 0.099 | 1.401 ± 0.094 |
| 23 | 1.15 | | 204.03 | L-Tryptophan | C11H12N2O2 | 203 | Amino acids | 0.031 ± 0.012 | 0.033 ± 0.013 | 0.036 ± 0.011 | 0.036 ± 0.014 | 0.022 ± 0.009 | 0.040 ± 0.016 | 0.027 ± 0.012 | 0.040 ± 0.012 |
| 24 | 1.23 | | 189.06 | Glutarylglycine | C7H11NO5 | 74, 144, 188 | Amino acids | 0.018 ± 0.007 | 0.010 ± 0.002 | 0.016 ± 0.005 | 0.008 ± 0.001 | 0.009 ± 0.004 | 0.010 ± 0.002 | 0.011 ± 0.004 | 0.011 ± 0.004 |
| 25 | 1.29 | | 174.20 | L-Theanine | C7H14N2O3 | 155, 177 | Amino acids | 3.785 ± 0.221 | 3.033 ± 0.209 | 2.799 ± 0.012 | 2.497 ± 0.063 | 3.262 ± 0.266 | 2.765 ± 0.215 | 2.672 ± 0.262 | 2.774 ± 0.186 |
| 26 | 1.29 | | 129.04 | Quinoline | C9H7N | 101, 128 | Alkaloids | 0.018 ± 0.005 | 0.021 ± 0.001 | 0.015 ± 0.004 | 0.019 ± 0.002 | 0.016 ± 0.005 | 0.017 ± 0.007 | 0.019 ± 0.003 | 0.016 ± 0.005 |
| 27 | 1.31 | | 464.12 | Hesperetin 7-glucoside | C22H24O11 | 131, 199 | Lipids | 0.007 ± 0.002 | 0.006 ± 0.001 | 0.006 ± 0.001 | 0.006 ± 0.001 | 0.004 ± 0.001 | 0.003 ± 0.001 | 0.003 ± 0.000 | 0.003 ± 0.000 |
| 28 | 1.31 | | 146.02 | Coumarin | C9H6O2 | 101, 145 | Alkaloids | 0.010 ± 0.001 | 0.015 ± 0.002 | 0.010 ± 0.002 | 0.013 ± 0.004 | 0.012 ± 0.001 | 0.016 ± 0.005 | 0.014 ± 0.003 | 0.013 ± 0.005 |
| 29 | 1.32 | | 329.07 | cAMP | C10H12N5O6P | 133 | Purine | 0.014 ± 0.005 | 0.021 ± 0.006 | 0.013 ± 0.005 | 0.018 ± 0.007 | 0.017 ± 0.006 | 0.015 ± 0.007 | 0.017 ± 0.006 | 0.013 ± 0.007 |
| 30 | 1.32 | | 307.08 | Glutathione | C10H17N3O6S | 128, 143, 179 | Amino acids, | 0.069 ± 0.011 | 0.082 ± 0.017 | 0.065 ± 0.001 | 0.084 ± 0.009 | 0.077 ± 0.004 | 0.076 ± 0.002 | 0.071 ± 0.005 | 0.071 ± 0.014 |
| 31 | 1.34 | | 204.03 | Daucic acid | C7H8O7 | 87, 115, 141, 203 | Organic acids | 0.160 ± 0.015 | 0.154 ± 0.013 | 0.147 ± 0.013 | 0.156 ± 0.015 | 0.169 ± 0.011 | 0.179 ± 0.023 | 0.159 ± 0.017 | 0.158 ± 0.012 |
| 32 | 1.35 | | 192.12 | Citric acid | C6H8O7 | 85, 87, 111 | Organic compounds | 1.743 ± 0.166 | 1.829 ± 0.176 | 1.730 ± 0.078 | 1.575 ± 0.133 | 1.683 ± 0.207 | 1.896 ± 0.099 | 1.756 ± 0.081 | 1.764 ± 0.097 |
| 33 | 1.37 | | 161.07 | Tryptophol | C10H11NO | 160 | Others | 0.006 ± 0.001 | 0.004 ± 0.001 | 0.004 ± 0.000 | 0.005 ± 0.000 | 0.006 ± 0.000 | 0.005 ± 0.001 | 0.005 ± 0.000 | 0.004 ± 0.000 |
| 34 | 1.43 | | 160.04 | Pimelic acid | C7H12O4 | 115, 158 | Organic acid | 0.006 ± 0.001 | 0.006 ± 0.001 | 0.005 ± 0.000 | 0.005 ± 0.000 | 0.003 ± 0.001 | 0.004 ± 0.001 | 0.003 ± 0.001 | 0.004 ± 0.001 |
| 35 | 1.46 | | 129.11 | L-Pyroglutamic acid | C5H7NO3 | 128, 129 | Flavonoid glycosides | 1.099 ± 0.015 | 1.046 ± 0.027 | 0.884 ± 0.041 | 0.878 ± 0.038 | 0.693 ± 0.038 | 0.547 ± 0.010 | 0.663 ± 0.070 | 0.731 ± 0.028 |
| 36 | 1.47 | | 134.06 | Malic acid | C4H6O5 | 71, 115, 133 | Organic acids | 0.018 ± 0.002 | 0.015 ± 0.001 | 0.013 ± 0.003 | 0.014 ± 0.001 | 0.008 ± 0.000 | 0.011 ± 0.000 | 0.014 ± 0.000 | 0.021 ± 0.002 |
| 37 | 1.61 | | 181.07 | L-Tyrosine | C9H11NO3 | 93, 119 | Amino acids, | 0.009 ± 0.001 | 0.008 ± 0.000 | 0.008 ± 0.000 | 0.008 ± 0.001 | 0.006 ± 0.001 | 0.006 ± 0.000 | 0.005 ± 0.001 | 0.007 ± 0.000 |
| 38 | 1.61 | | 190.05 | Cytisine | C11H14N2O | 130, 189 | Alkaloids | 0.003 ± 0.001 | 0.004 ± 0.001 | 0.003 ± 0.000 | 0.004 ± 0.001 | 0.003 ± 0.000 | 0.003 ± 0.001 | 0.003 ± 0.000 | 0.004 ± 0.001 |
| 39 | 1.69 | | 302.00 | Ellagic acid | C14H6O8 | 300 | Organic acid | 0.001 ± 0.000 | 0.002 ± 0.000 | 0.002 ± 0.000 | 0.002 ± 0.000 | 0.003 ± 0.000 | 0.003 ± 0.000 | 0.003 ± 0.000 | 0.003 ± 0.000 |
| 40 | 1.76 | | 332.26 | Galloylglucose | C13H16O10 | 125, 169, 331 | Organic compounds | 0.862 ± 0.079 | 1.032 ± 0.017 | 0.716 ± 0.063 | 0.717 ± 0.003 | 0.733 ± 0.030 | 0.734 ± 0.007 | 0.806 ± 0.012 | 0.961 ± 0.054 |
| 41 | 1.84 | | 162.05 | Methyl cinnamate | C10H10O2 | 101, 103 | Organic acid | 0.001 ± 0.000 | 0.001 ± 0.000 | 0.001 ± 0.000 | 0.001 ± 0.000 | 0.005 ± 0.000 | 0.004 ± 0.001 | 0.004 ± 0.001 | 0.004 ± 0.001 |
| 42 | 2.13 | | 216.11 | Curzerene | C15H20O | 133, 173, 215 | Lipids | 0.008 ± 0.001 | 0.008 ± 0.000 | 0.008 ± 0.000 | 0.007 ± 0.000 | 0.010 ± 0.001 | 0.009 ± 0.001 | 0.009 ± 0.000 | 0.008 ± 0.001 |
| 43 | 2.14 | | 170.12 | 2, 3, 4-Trihydroxybenzoic acid | C7H6O5 | 69, 91, 107, 125 | Organic compounds | 1.135 ± 0.054 | 1.113 ± 0.042 | 1.002 ± 0.095 | 0.971 ± 0.050 | 0.887 ± 0.100 | 0.790 ± 0.063 | 0.850 ± 0.012 | 0.806 ± 0.088 |
| 44 | 2.24 | | 366.06 | Glycyrol | C21H18O6 | 321, 365 | Isoflavonoids | 0.711 ± 0.046 | 0.557 ± 0.025 | 0.487 ± 0.033 | 0.516 ± 0.033 | 0.687 ± 0.035 | 0.556 ± 0.013 | 0.585 ± 0.025 | 0.490 ± 0.024 |
| 45 | 2.52 | | 332.07 | 1-O-Galloyl-β-D-glucose | C13H16O10 | 125, 169 | Organic acid | 0.005 ± 0.002 | 0.001 ± 0.001 | 0.003 ± 0.001 | 0.006 ± 0.001 | 0.007 ± 0.000 | 0.008 ± 0.001 | 0.007 ± 0.002 | 0.007 ± 0.001 |
| 46 | 2.71 | | 165.08 | L-Phenylalanine | C9H11NO2 | 147 | Amino acids, | 0.011 ± 0.001 | 0.009 ± 0.001 | 0.011 ± 0.001 | 0.011 ± 0.000 | 0.008 ± 0.000 | 0.008 ± 0.000 | 0.006 ± 0.000 | 0.008 ± 0.000 |
| 47 | 2.97 | | 420.09 | Morusin | C25H24O6 | 91, 267 | Flavonoids | 0.003 ± 0.000 | 0.004 ± 0.000 | 0.004 ± 0.000 | 0.003 ± 0.000 | 0.001 ± 0.000 | 0.002 ± 0.000 | 0.002 ± 0.000 | 0.002 ± 0.000 |
| 48 | 3.28 | | 202.13 | Spermine | C10H26N4 | 129, 201 | Others | 0.000 ± 0.000 | 0.000 ± 0.000 | 0.000 ± 0.000 | 0.000 ± 0.000 | 0.001 ± 0.000 | 0.001 ± 0.000 | 0.001 ± 0.000 | 0.001 ± 0.000 |
| 49 | 3.67 | | 306.07 | Gallocatechin | C15H14O7 | 125, 305 | Flavonoids | 0.002 ± 0.002 | 0.004 ± 0.003 | 0.000 ± 0.000 | 0.001 ± 0.001 | 0.012 ± 0.014 | 0.001 ± 0.002 | 0.000 ± 0.000 | 0.002 ± 0.002 |
| 50 | 3.81 | | 306.27 | (+)-Gallocatechin | C15H14O7 | 167, 179, 219 | Catechins | 0.557 ± 0.048 | 0.650 ± 0.043 | 0.840 ± 0.035 | 0.891 ± 0.079 | 0.790 ± 0.055 | 0.736 ± 0.012 | 0.785 ± 0.061 | 0.770 ± 0.045 |
| 51 | 4.61 | | 135.05 | Adenine | C5H5N5 | 107, 134 | Alkaloids | 0.002 ± 0.001 | 0.002 ± 0.000 | 0.002 ± 0.000 | 0.002 ± 0.000 | 0.001 ± 0.000 | 0.002 ± 0.000 | 0.002 ± 0.000 | 0.002 ± 0.000 |
| 52 | 4.88 | | 340.08 | Esculin | C15H16O9 | 151, 177 | Carbohydrates | 0.001 ± 0.000 | 0.001 ± 0.000 | 0.001 ± 0.000 | 0.001 ± 0.000 | 0.001 ± 0.000 | 0.002 ± 0.000 | 0.002 ± 0.000 | 0.002 ± 0.000 |
| 53 | 4.99 | | 126.03 | Maltol | C6H6O3 | 125 | Others | 0.001 ± 0.000 | 0.001 ± 0.000 | 0.001 ± 0.000 | 0.002 ± 0.000 | 0.003 ± 0.000 | 0.003 ± 0.000 | 0.003 ± 0.001 | 0.003 ± 0.000 |
| 54 | 5.14 | | 294.13 | [6]-Gingerol | C17H26O4 | 59, 71, 119, 293 | Organic acid | 0.006 ± 0.000 | 0.007 ± 0.002 | 0.006 ± 0.001 | 0.005 ± 0.000 | 0.005 ± 0.000 | 0.006 ± 0.001 | 0.006 ± 0.000 | 0.005 ± 0.000 |
| 55 | 5.27 | | 634.45 | Sanguiin H4 | C27H22O18 | 169, 463 | Organic compounds | 1.241 ± 0.053 | 1.453 ± 0.103 | 1.420 ± 0.032 | 1.508 ± 0.035 | 1.320 ± 0.069 | 1.462 ± 0.048 | 1.489 ± 0.053 | 1.509 ± 0.050 |
| 56 | 5.30 | | 290.27 | (+)-Epicatechin | C15H14O6 | 125, 137, 179, 245 | Catechins | 0.440 ± 0.016 | 0.493 ± 0.023 | 0.513 ± 0.002 | 0.517 ± 0.031 | 0.446 ± 0.001 | 0.327 ± 0.003 | 0.445 ± 0.050 | 0.470 ± 0.058 |
| 57 | 5.31 | | 422.34 | Mangiferin | C19H18O11 | 217, 375 | Flavonoids | 0.005 ± 0.002 | 0.005 ± 0.000 | 0.004 ± 0.000 | 0.007 ± 0.001 | 0.013 ± 0.000 | 0.014 ± 0.001 | 0.010 ± 0.000 | 0.010 ± 0.001 |
| 58 | 5.31 | | 376.36 | Riboflavin | C17H20N4O6 | 243, 375 | Flavonoids | 0.007 ± 0.002 | 0.007 ± 0.001 | 0.006 ± 0.000 | 0.009 ± 0.001 | 0.017 ± 0.001 | 0.018 ± 0.001 | 0.014 ± 0.001 | 0.013 ± 0.002 |
| 59 | 5.32 | | 184.15 | Methyl gallate | C8H8O5 | 111, 183 | Organic compounds | 0.021 ± 0.003 | 0.021 ± 0.003 | 0.016 ± 0.002 | 0.013 ± 0.000 | 0.012 ± 0.010 | 0.013 ± 0.005 | 0.009 ± 0.004 | 0.016 ± 0.008 |
| 60 | 5.34 | | 350.19 | (1S, 2R, 4R, 8S)-p-Menthane-2, 8, 9-triol 9-glucoside | C16H30O8 | 89, 161, 349 | Lipids | 0.012 ± 0.005 | 0.017 ± 0.006 | 0.018 ± 0.004 | 0.010 ± 0.002 | 0.010 ± 0.004 | 0.008 ± 0.000 | 0.007 ± 0.001 | 0.023 ± 0.005 |
| 61 | 5.36 | | 286.05 | Luteolin | C15H10O6 | 121, 133 | Flavonoids | 0.003 ± 0.000 | 0.003 ± 0.000 | 0.004 ± 0.000 | 0.004 ± 0.000 | 0.005 ± 0.000 | 0.003 ± 0.000 | 0.004 ± 0.000 | 0.003 ± 0.000 |
| 62 | 5.44 | | 634.08 | Punicacortein B | C20H22N6O16S | 75, 125, 169 | Others | 0.111 ± 0.002 | 0.072 ± 0.027 | 0.124 ± 0.008 | 0.130 ± 0.007 | 0.077 ± 0.033 | 0.120 ± 0.003 | 0.134 ± 0.008 | 0.135 ± 0.005 |
| 63 | 5.50 | | 318.05 | Myricetin | C15H10O8 | 125, 151, 271 | Flavonoids | 0.002 ± 0.000 | 0.002 ± 0.000 | 0.003 ± 0.000 | 0.002 ± 0.000 | 0.001 ± 0.000 | 0.001 ± 0.000 | 0.001 ± 0.000 | 0.002 ± 0.000 |
| 64 | 5.73 | | 338.16 | Bergamottin | C21H22O4 | 119, 173, 191 | Lipids | 0.007 ± 0.000 | 0.007 ± 0.000 | 0.007 ± 0.000 | 0.007 ± 0.000 | 0.008 ± 0.000 | 0.009 ± 0.000 | 0.008 ± 0.000 | 0.009 ± 0.000 |
| 65 | 5.79 | | 388.17 | Verbenalin | C17H24O10 | 59, 163, 207 | Lipids | 0.016 ± 0.000 | 0.014 ± 0.000 | 0.016 ± 0.001 | 0.020 ± 0.001 | 0.014 ± 0.000 | 0.022 ± 0.000 | 0.020 ± 0.001 | 0.022 ± 0.001 |
| 66 | 5.83 | | 164.05 | 2-Hydroxycinnamic acid | C9H8O3 | 91, 119 | Organic acid | 0.002 ± 0.001 | 0.002 ± 0.000 | 0.002 ± 0.000 | 0.001 ± 0.000 | 0.003 ± 0.001 | 0.003 ± 0.000 | 0.003 ± 0.000 | 0.003 ± 0.001 |
| 67 | 5.88 | | 174.05 | Shikimic acid | C7H10O5 | 93, 173 | Organic acid | 0.006 ± 0.003 | 0.009 ± 0.001 | 0.005 ± 0.003 | 0.003 ± 0.003 | 0.016 ± 0.006 | 0.005 ± 0.006 | 0.003 ± 0.003 | 0.003 ± 0.003 |
| 68 | 5.90 | | 126.03 | Thymine | C5H6N2O2 | 107, 125 | Others | 0.058 ± 0.002 | 0.067 ± 0.001 | 0.076 ± 0.004 | 0.077 ± 0.004 | 0.065 ± 0.006 | 0.070 ± 0.002 | 0.077 ± 0.002 | 0.080 ± 0.001 |
| 69 | 5.90 | | 170.02 | 2, 4, 6-Trihydroxybenzoic acid | C7H6O5 | 125, 169 | Organic acid | 0.111 ± 0.003 | 0.117 ± 0.004 | 0.126 ± 0.004 | 0.135 ± 0.005 | 0.119 ± 0.009 | 0.128 ± 0.004 | 0.132 ± 0.005 | 0.107 ± 0.050 |
| 70 | 5.95 | | 338.10 | 3-O-p-Coumaroylquinic acid | C16H18O8 | 163, 173, 191 | Organic acid | 0.291 ± 0.012 | 0.271 ± 0.031 | 0.233 ± 0.012 | 0.230 ± 0.008 | 0.607 ± 0.004 | 0.416 ± 0.007 | 0.397 ± 0.018 | 0.438 ± 0.016 |
| 71 | 5.96 | | 154.03 | Protocatechuic acid | C7H6O4 | 109, 153 | Organic acids | 0.015 ± 0.002 | 0.012 ± 0.000 | 0.010 ± 0.001 | 0.011 ± 0.001 | 0.015 ± 0.001 | 0.018 ± 0.000 | 0.018 ± 0.001 | 0.014 ± 0.001 |
| 72 | 6.05 | | 320.05 | (+)-Dihydromyricetin | C15H12O8 | 125, 193, 301 | Flavonoids | 0.029 ± 0.002 | 0.029 ± 0.001 | 0.032 ± 0.002 | 0.030 ± 0.000 | 0.026 ± 0.000 | 0.024 ± 0.000 | 0.024 ± 0.001 | 0.027 ± 0.001 |
| 73 | 6.07 | | 478.08 | Chrysoeriol 7-O-(6''-malonyl-glucoside) | C21H18O13 | 61, 299 | Flavonoids | 0.034 ± 0.004 | 0.026 ± 0.004 | 0.053 ± 0.006 | 0.066 ± 0.008 | 0.030 ± 0.002 | 0.055 ± 0.048 | 0.052 ± 0.045 | 0.086 ± 0.012 |
| 74 | 6.08 | | 772.21 | Quercetin 3-O-glucosyl-rutinoside | C33H40O21 | 15, 1271, 301 | Flavonoids | 0.013 ± 0.009 | 0.016 ± 0.022 | 0.021 ± 0.005 | 0.031 ± 0.027 | 0.033 ± 0.002 | 0.012 ± 0.013 | 0.053 ± 0.025 | 0.028 ± 0.019 |
| 75 | 6.11 | | 564.49 | Theaflavin | C29H24O12 | 125, 563 | Flavonoid glycosides | 0.016 ± 0.001 | 0.015 ± 0.001 | 0.016 ± 0.001 | 0.017 ± 0.001 | 0.026 ± 0.002 | 0.042 ± 0.002 | 0.034 ± 0.001 | 0.034 ± 0.002 |
| 76 | 6.12 | | 594.16 | Vicenin 2 | C27H30O15 | 311, 473 | Flavonoids | 0.002 ± 0.000 | 0.002 ± 0.000 | 0.001 ± 0.000 | 0.001 ± 0.000 | 0.001 ± 0.000 | 0.001 ± 0.000 | 0.001 ± 0.000 | 0.001 ± 0.000 |
| 77 | 6.22 | | 464.10 | Isoquercitrin | C21H20O12 | 300 | Flavonoids | 0.001 ± 0.000 | 0.001 ± 0.000 | 0.001 ± 0.000 | 0.001 ± 0.000 | 0.001 ± 0.000 | 0.001 ± 0.000 | 0.001 ± 0.000 | 0.001 ± 0.000 |
| 78 | 6.33 | | 772.66 | Kaempferol 7-(3G-glucosylgentiobioside) | C33H40O21 | 151, 179, 327 | Flavonoid glycosides | 0.139 ± 0.016 | 0.141 ± 0.021 | 0.141 ± 0.013 | 0.148 ± 0.007 | 0.179 ± 0.014 | 0.177 ± 0.009 | 0.189 ± 0.018 | 0.230 ± 0.001 |
| 79 | 6.35 | | 226.12 | (+)-Genipin | C11H14O5 | 59 | Lipids | 0.007 ± 0.000 | 0.007 ± 0.000 | 0.007 ± 0.000 | 0.008 ± 0.000 | 0.011 ± 0.000 | 0.009 ± 0.000 | 0.010 ± 0.001 | 0.011 ± 0.001 |
| 80 | 6.54 | | 210.09 | Sinapyl alcohol | C11H14O4 | 181, 209 | Organic acid | 0.006 ± 0.001 | 0.006 ± 0.000 | 0.005 ± 0.000 | 0.005 ± 0.000 | 0.004 ± 0.000 | 0.004 ± 0.000 | 0.004 ± 0.000 | 0.004 ± 0.000 |
| 81 | 6.55 | | 610.52 | Rutin | C27H30O16 | 609 | Flavonoid glycosides | 0.046 ± 0.002 | 0.052 ± 0.005 | 0.042 ± 0.002 | 0.052 ± 0.003 | 0.073 ± 0.002 | 0.060 ± 0.001 | 0.065 ± 0.006 | 0.054 ± 0.001 |
| 82 | 6.65 | | 432.11 | Vitexin | C21H20O10 | 283, 311, 341 | Flavonoids | 0.005 ± 0.000 | 0.006 ± 0.000 | 0.006 ± 0.000 | 0.006 ± 0.000 | 0.006 ± 0.000 | 0.012 ± 0.000 | 0.009 ± 0.001 | 0.009 ± 0.001 |
| 83 | 6.68 | | 756.66 | Kaempferol 3-O-glucosyl-rhamnosyl-glucoside | C33H40O20 | 285, 327, 755 | Flavonoid glycosides | 0.218 ± 0.005 | 0.202 ± 0.006 | 0.226 ± 0.010 | 0.251 ± 0.003 | 0.300 ± 0.023 | 0.406 ± 0.004 | 0.347 ± 0.018 | 0.283 ± 0.013 |
| 84 | 6.71 | | 464.38 | Quercetin 3-galactoside | C21H20O12 | 300, 463 | Flavonoids | 0.086 ± 0.006 | 0.079 ± 0.011 | 0.080 ± 0.006 | 0.077 ± 0.005 | 0.115 ± 0.003 | 0.104 ± 0.004 | 0.108 ± 0.008 | 0.094 ± 0.013 |
| 85 | 6.80 | | 448.10 | Astragalin | C21H20O11 | 125, 153, 267, 285 | Flavonoids | 0.003 ± 0.000 | 0.003 ± 0.000 | 0.003 ± 0.000 | 0.003 ± 0.000 | 0.002 ± 0.000 | 0.003 ± 0.000 | 0.003 ± 0.000 | 0.003 ± 0.000 |
| 86 | 6.81 | | 594.16 | Pelargonidin 3, 5-di-O-glucoside | C28H26N4O11 | 87, 105, 594 | Flavonoid glycosides | 0.007 ± 0.000 | 0.007 ± 0.000 | 0.006 ± 0.000 | 0.007 ± 0.000 | 0.010 ± 0.000 | 0.024 ± 0.002 | 0.021 ± 0.001 | 0.013 ± 0.001 |
| 87 | 6.93 | | 595.53 | Cyanidin 3-rutinoside | C26H31N2O12P | 125, 285, 593 | Flavonoid glycosides | 0.032 ± 0.004 | 0.034 ± 0.001 | 0.040 ± 0.003 | 0.047 ± 0.002 | 0.057 ± 0.003 | 0.086 ± 0.003 | 0.061 ± 0.002 | 0.050 ± 0.003 |
| 88 | 7.04 | | 449.38 | Cyanidin 3-glucoside | C20H21N2O8P | 255, 286, 447 | Flavonoid glycosides | 0.149 ± 0.003 | 0.173 ± 0.011 | 0.186 ± 0.004 | 0.176 ± 0.003 | 0.157 ± 0.007 | 0.249 ± 0.017 | 0.240 ± 0.016 | 0.257 ± 0.006 |
| 89 | 7.04 | | 434.09 | Avicularin | C20H18O11 | 151, 271 | Flavonoids | 0.005 ± 0.000 | 0.005 ± 0.000 | 0.006 ± 0.000 | 0.006 ± 0.000 | 0.004 ± 0.000 | 0.005 ± 0.000 | 0.006 ± 0.000 | 0.007 ± 0.000 |
| 90 | 7.17 | | 466.44 | Agnuside | C22H26O11 | 151, 303 | Lipids | 0.147 ± 0.003 | 0.170 ± 0.011 | 0.182 ± 0.004 | 0.089 ± 0.057 | 0.081 ± 0.011 | 0.136 ± 0.005 | 0.144 ± 0.065 | 0.140 ± 0.084 |
| 91 | 7.17 | | 449.38 | Cyanidin 3-galactoside | C22H16N4O7 | 125, 227, 286, 447 | Flavonoid glycosides | 0.046 ± 0.004 | 0.054 ± 0.004 | 0.057 ± 0.005 | 0.056 ± 0.012 | 0.080 ± 0.011 | 0.136 ± 0.005 | 0.099 ± 0.006 | 0.088 ± 0.011 |
| 92 | 7.17 | | 274.27 | Phloretin | C15H14O5 | 161, 273 | Flavonoids | 0.023 ± 0.003 | 0.058 ± 0.001 | 0.056 ± 0.001 | 0.058 ± 0.004 | 0.005 ± 0.001 | 0.009 ± 0.002 | 0.022 ± 0.007 | 0.027 ± 0.015 |
| 93 | 8.39 | | 264.14 | Abscisic acid | C15H20O4 | 139, 201, 219 | Lipids | 0.010 ± 0.000 | 0.011 ± 0.000 | 0.013 ± 0.001 | 0.013 ± 0.000 | 0.011 ± 0.000 | 0.010 ± 0.000 | 0.011 ± 0.000 | 0.012 ± 0.000 |
| 94 | 8.67 | | 507.42 | Delphinidin 3-(acetylglucoside) | C24H14N10O4 | 70, 89, 161, 506 | Flavonoid glycosides | 0.005 ± 0.000 | 0.006 ± 0.000 | 0.005 ± 0.001 | 0.007 ± 0.000 | 0.011 ± 0.000 | 0.014 ± 0.001 | 0.015 ± 0.001 | 0.017 ± 0.001 |
| 95 | 8.76 | | 272.07 | Naringenin | C15H12O5 | 119, 151 | Flavanones | 0.004 ± 0.000 | 0.006 ± 0.001 | 0.003 ± 0.002 | 0.007 ± 0.000 | 0.003 ± 0.001 | 0.003 ± 0.001 | 0.006 ± 0.000 | 0.006 ± 0.000 |
| 96 | 8.82 | | 286.05 | Kaempferol | C15H10O6 | 285 | Flavones | 0.010 ± 0.002 | 0.021 ± 0.003 | 0.023 ± 0.002 | 0.027 ± 0.001 | 0.009 ± 0.003 | 0.012 ± 0.004 | 0.021 ± 0.001 | 0.024 ± 0.001 |
| 97 | 8.89 | | 330.24 | 17-Hydroxyprogesterone | C21H30O3 | 229, 329 | Lipids | 0.003 ± 0.000 | 0.003 ± 0.000 | 0.003 ± 0.000 | 0.003 ± 0.000 | 0.002 ± 0.000 | 0.003 ± 0.000 | 0.003 ± 0.000 | 0.003 ± 0.000 |
| 98 | 9.23 | | 188.14 | ε-Acetyl-L-lysine | C8H16N2O3 | 141, 187 | Amino acids | 0.002 ± 0.000 | 0.001 ± 0.000 | 0.001 ± 0.000 | 0.001 ± 0.000 | 0.002 ± 0.000 | 0.001 ± 0.000 | 0.001 ± 0.000 | 0.001 ± 0.000 |
| 99 | 9.77 | | 243.09 | Cytidine | C9H13N3O5 | 242 | Nucleosides | 0.001 ± 0.000 | 0.001 ± 0.000 | 0.001 ± 0.000 | 0.001 ± 0.000 | 0.002 ± 0.000 | 0.002 ± 0.000 | 0.002 ± 0.000 | 0.002 ± 0.000 |
| 100 | 10.77 | | 324.16 | Escitalopram | C8H24N10S2 | 238, 307, 323 | Organic acids | 0.109 ± 0.061 | 0.007 ± 0.007 | 0.012 ± 0.002 | 0.005 ± 0.003 | 0.078 ± 0.046 | 0.069 ± 0.042 | 0.005 ± 0.002 | 0.004 ± 0.002 |
| 101 | 11.74 | | 166.03 | 3-Methylxanthine | C6H6N4O2 | 149 | Purines | 0.001 ± 0.000 | 0.001 ± 0.000 | 0.001 ± 0.000 | 0.001 ± 0.000 | 0.001 ± 0.000 | 0.001 ± 0.000 | 0.001 ± 0.000 | 0.001 ± 0.000 |

Table S3 Taste addition experiment of umami-contributors

| No. | Formula | Umami score |
| --- | --- | --- |
| 1 | 182.12 mg/L *L*-Aspartic acid | 1.75 ± 0.25 |
| 2 | 13.04 mg/L *L*-Arginine | 1.43 ± 0.38 |
| 3 | 1175.35 mg/L *L*-Theanine | 2.13 ± 0.26 |

Table S4 Correlation analysis of taste quality attributes with key non-volatile compounds.

| Non-volatile compounds | Bitterness | Astringency | Umami | Umami-mellow taste | Overall taste | Temperature |
| --- | --- | --- | --- | --- | --- | --- |
| *L*-Pyroglutamic acid | 0.300 | 0.287 | 0.749^**^ | 0.403 | 0.487^*^ | -0.182 |
| *L*-Aspartic acid | -0.138 | -0.136 | 0.565^**^ | 0.533^**^ | 0.437^*^ | -0.422^*^ |
| *D*-Malic acid | -0.166 | -0.168 | -0.566^**^ | -0.104 | -0.298 | 0.154 |
| Hypoxanthine | -0.392 | -0.316 | -0.288 | 0.172 | -0.125 | -0.225 |
| *L*-Arabinose | -0.112 | -0.091 | 0.363 | 0.262 | 0.264 | -0.286 |
| 7-Methylxanthine | -0.342 | -0.292 | 0.614^**^ | 0.757^**^ | 0.562^**^ | -0.681^**^ |
| *L*-Theanine | -0.448^*^ | -0.493^*^ | 0.465^*^ | 0.790^**^ | 0.547^**^ | -0.733^**^ |
| *L*-Arginine | -0.045 | -0.019 | 0.702^**^ | 0.651^**^ | 0.599^**^ | -0.469^*^ |
| *L*-Ascorbic acid | -0.004 | -0.002 | -0.606^**^ | -0.308 | -0.311 | 0.328 |
| Methyl gallate | 0.147 | 0.118 | 0.205 | 0.189 | 0.113 | -0.189 |
| Citric acid | -0.192 | -0.154 | -0.023 | 0.320 | 0.074 | -0.17 |
| Quinic acid | -0.535^**^ | -0.552^**^ | -0.402 | 0.051 | -0.167 | -0.224 |
| Phloretin | 0.741^**^ | 0.706^**^ | 0.379 | -0.288 | 0.16 | 0.483^*^ |
| (+)-Epicatechin | 0.542^**^ | 0.432^*^ | 0.251 | -0.247 | 0.155 | 0.379 |
| (+)-Gallocatechin | 0.283 | 0.371 | -0.406^*^ | -0.776^**^ | -0.403 | 0.595^**^ |
| 5, 7, 4′-Trimethoxyflavone | 0.189 | 0.002 | 0.142 | 0.001 | 0.209 | -0.069 |
| Galloylglucose | 0.207 | 0.165 | 0.077 | 0.130 | -0.147 | 0.002 |
| Riboflavin | -0.605^**^ | -0.574^**^ | -0.631^**^ | -0.057 | -0.311 | -0.172 |
| Mangiferin | -0.555^**^ | -0.507^*^ | -0.606^**^ | -0.108 | -0.356 | -0.091 |
| Cyanidin 3-galactoside | -0.384 | -0.259 | -0.645^**^ | -0.229 | -0.460^*^ | 0.044 |
| Cyanidin 3-glucoside | 0.186 | 0.202 | -0.734^**^ | -0.515^**^ | -0.657^**^ | 0.511^*^ |
| Quercetin 3-galactoside | -0.630^**^ | -0.543^**^ | -0.501^*^ | 0.020 | -0.308 | -0.305 |
| Agnuside | 0.081 | 0.413^*^ | 0.128 | -0.014 | -0.220 | 0.023 |
| Delphinidin 3-(acetylglucoside) | -0.121 | -0.082 | -0.848^**^ | -0.470^*^ | -0.761^**^ | 0.317 |
| Theaflavin | -0.289 | -0.222 | -0.719^**^ | -0.265 | -0.566^**^ | 0.138 |
| Cyanidin 3-rutinoside | -0.416^*^ | -0.343 | -0.615^**^ | -0.194 | -0.329 | 0.024 |
| Rutin | -0.586^**^ | -0.515^**^ | -0.452^*^ | -0.111 | -0.332 | -0.236 |
| Sanguiin H4 | 0.428^*^ | 0.495^*^ | -0.440^*^ | -0.651^**^ | -0.498^*^ | 0.705^**^ |
| Kaempferol 3-*O*-glucosyl-rhamnosyl-glucoside | -0.404 | -0.344 | -0.652^**^ | -0.202 | -0.381 | 0.013 |
| Kaempferol 7-(3*G*-glucosylgentiobioside) | -0.038 | -0.014 | -0.772^**^ | -0.508^*^ | -0.763^**^ | 0.335 |


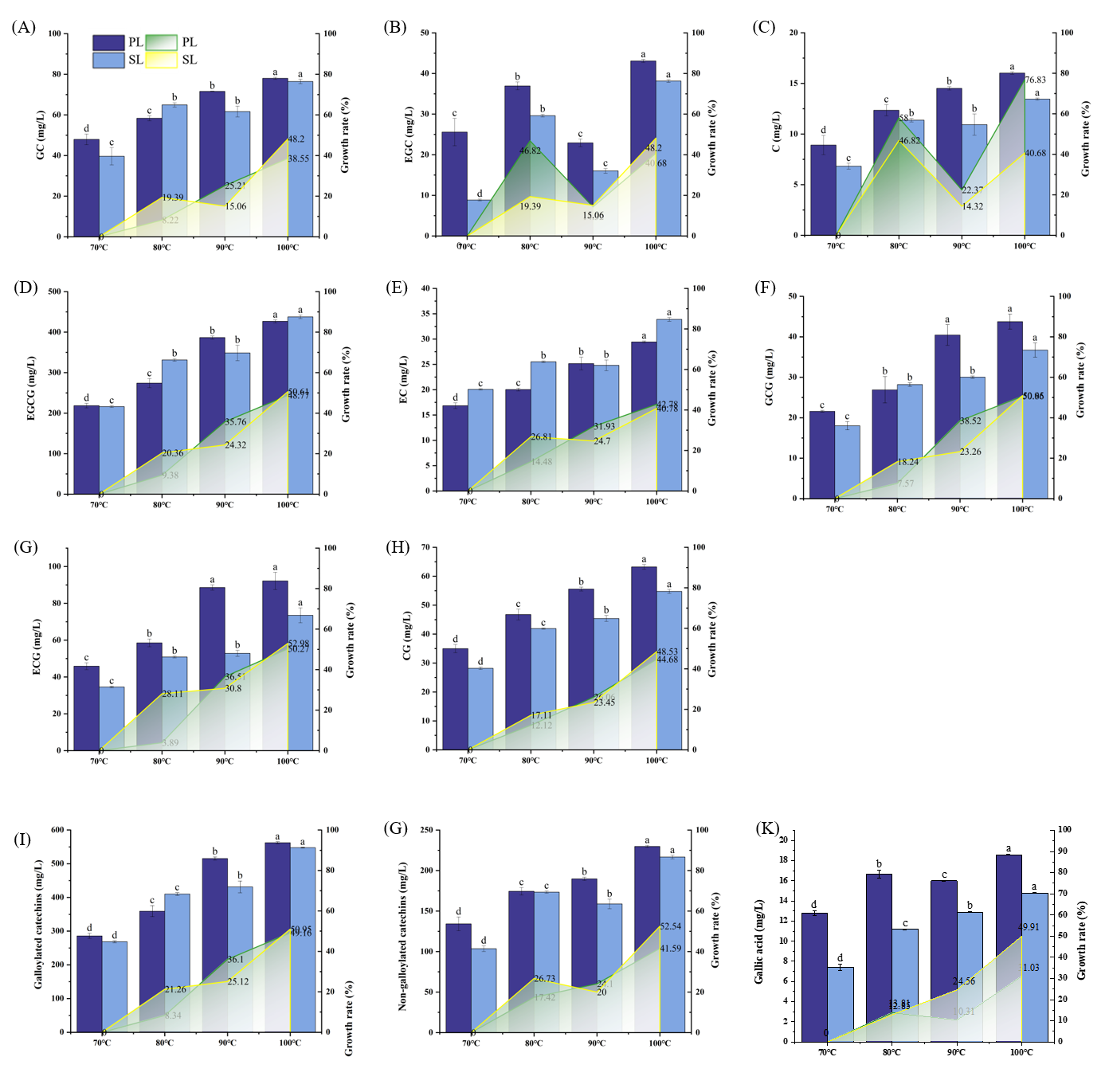


**Fig. S1. The contents of catechins in tea infusions.** PL: Premium Longjing tea; SL: Standard Longjing tea.


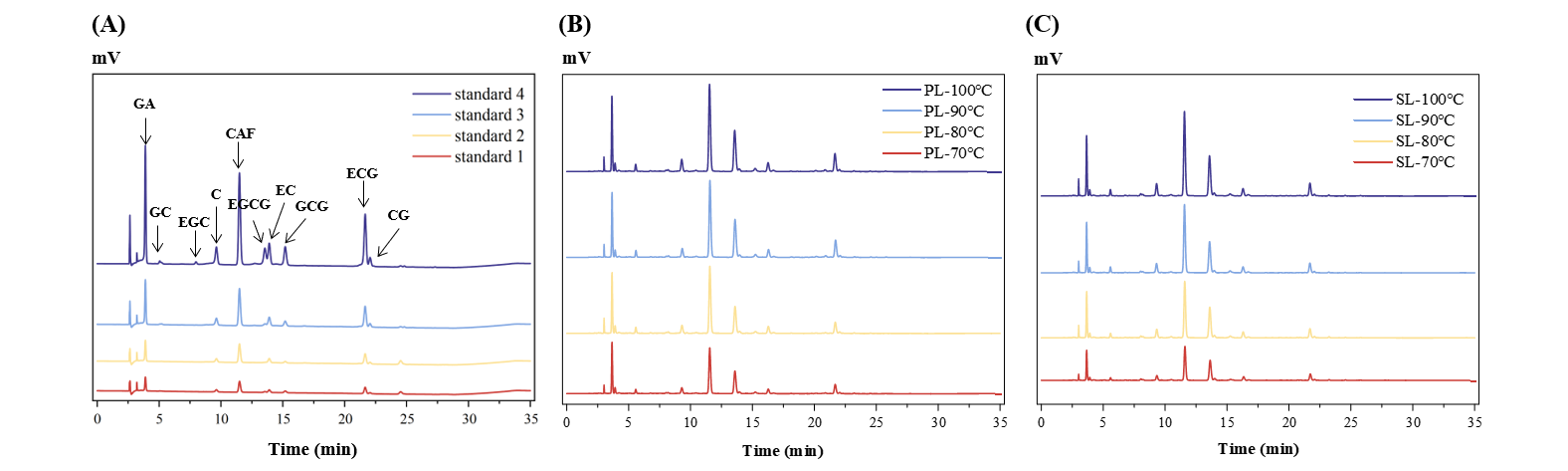


**Fig. S2. Typical HPLC chromatogram.** PL: Premium Longjing tea; SL: Standard Longjing tea.


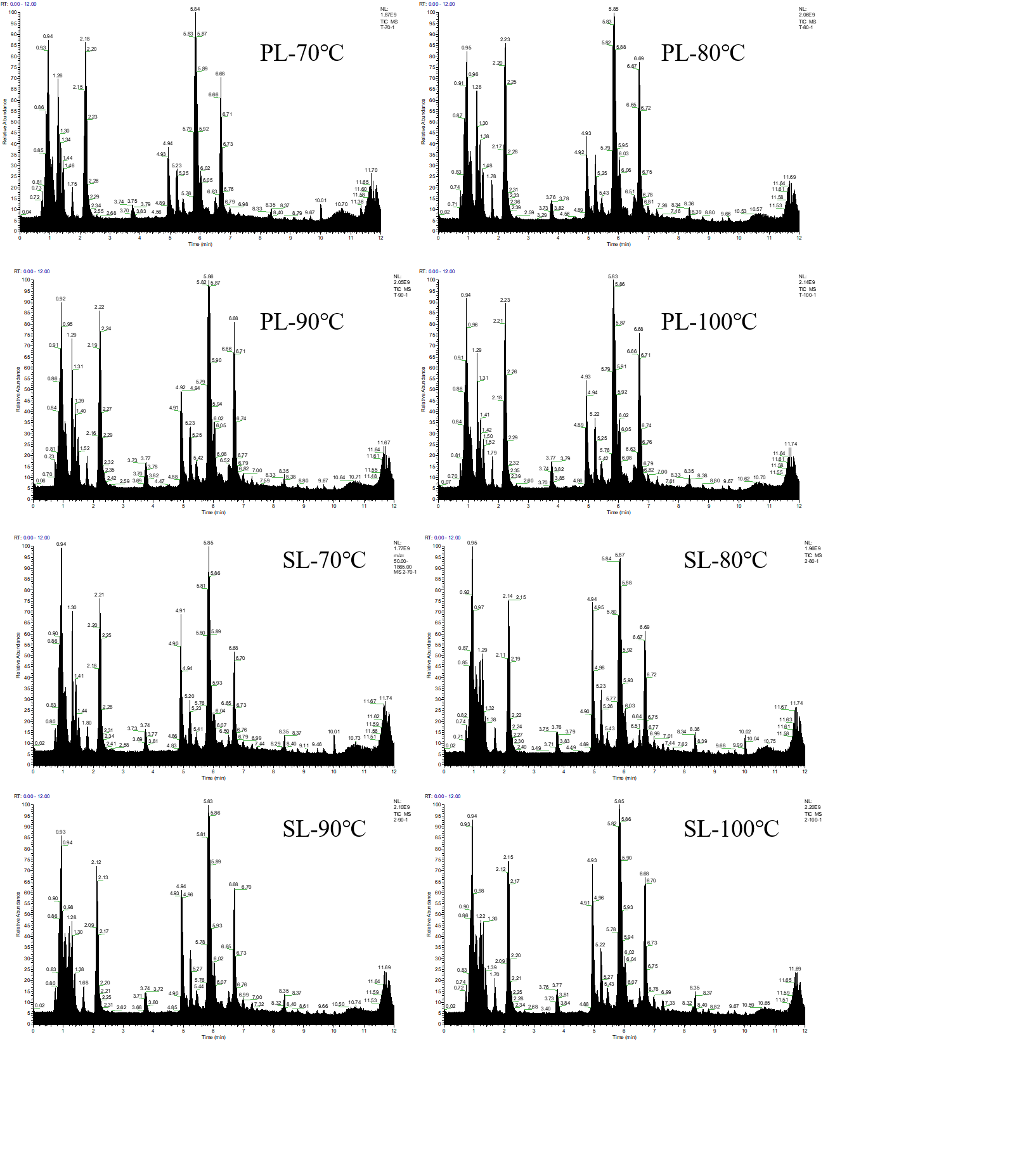


**Fig. S3. Typical LCMS chromatogram.** PL: Premium Longjing tea; SL: Standard Longjing tea.


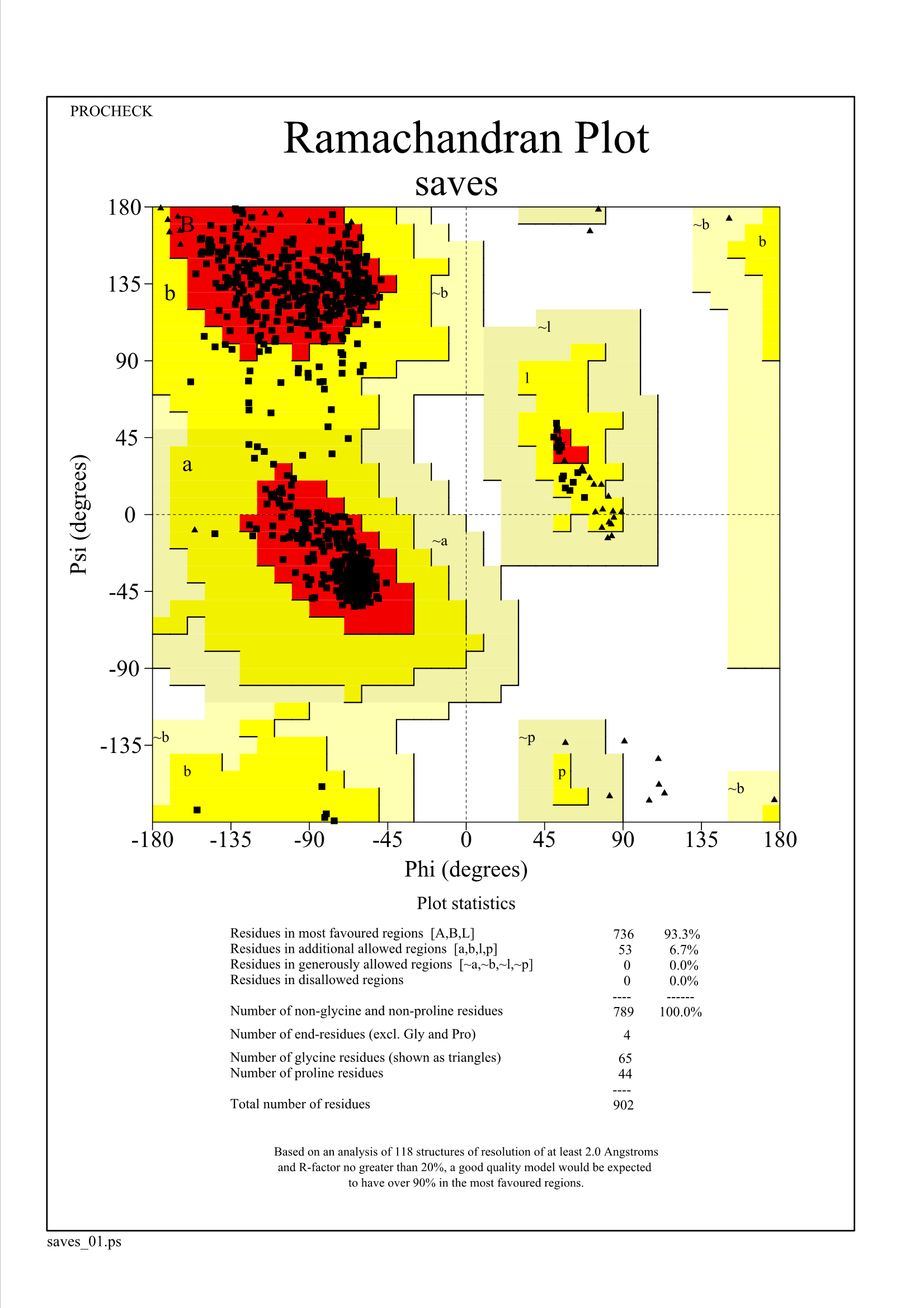


**Fig. S4. Ramachandran plot for the T1R1/T1R3 prediction model.**
